# Supplementary material for: A tightly-bonded and flexible mesoporous zeolite-cotton hybrid hemostat
Source: Nat Commun. 2019 Apr 29;10:1932. doi: 10.1038/s41467-019-09849-9 (PMC6488602; doi:10.1038/s41467-019-09849-9)
Supplement: Supplementary file 1 — Supplementary Information [file 41467_2019_9849_MOESM1_ESM.pdf]

A Tightly-Bonded and Flexible Mesoporous Zeolite-Cotton Hybrid Hemostat

Yu et al.

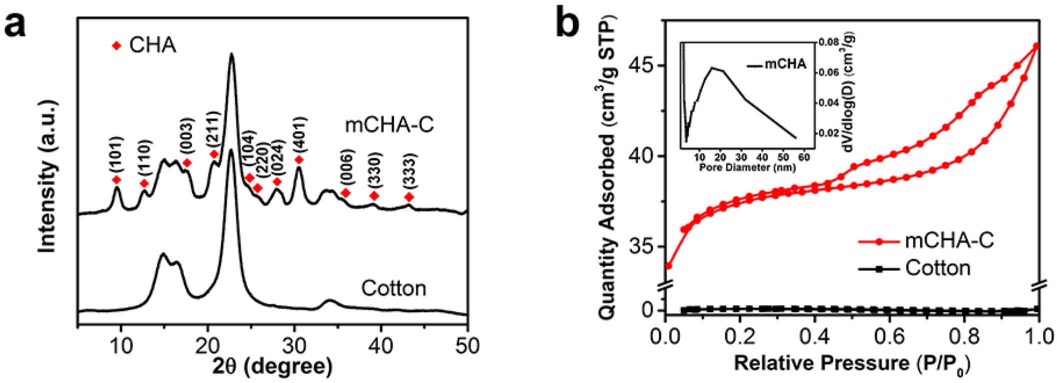

Supplementary Figure 1. (a) XRD patterns of mCHA-C and cotton. Source data are provided as a Source Data file. **(b)** Nitrogen sorption isotherm of mCHA-C and cotton, and pore size distribution of mCHA zeolite (inset). Source data are provided as a Source Data file.

Supplementary Table 1

The mCHA content on the cotton.

Sample	1	2	3	Mean	Standard Deviation
mCHA Content (wt%) [#]	22.5	23.5	24.0	23.3	0.8

[#] The mCHA content was measured by thermogravimetry analysis (TGA).

Supplementary Table 2

Nitrogen sorption data of cotton, mCHA-C, and mCHA zeolite.

Samples	BET surface area (m^2 g^{-1})	Micropore volume (m^3 g^{-1})	Mesopore volume (m^3 g^{-1})
Cotton	1.0	-	-
mCHA-C	114	0.052	0.019
mCHA [#]	489	0.22	0.08

[#] The cotton itself is nonporous, which shows a negligible surface areas (1.0 m^2 g^{-1}). Nitrogen sorption data of mCHA zeolite was estimated by subtracting the sorption from cotton.

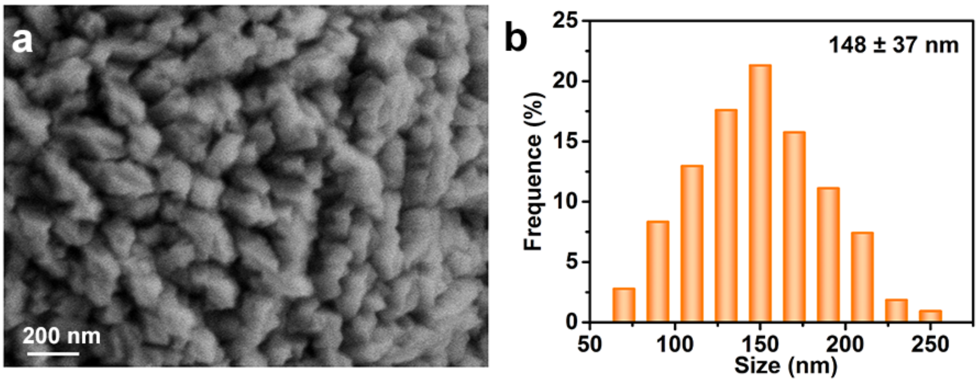

Supplementary Figure 2. (a) FE-SEM image of mCHA on the cotton. **(b)** Size distribution of CHA nanocrystals observed by FE-SEM images. Source data are provided as a Source Data file. Each sphere CHA is consisted of nanocrystals with a size of 148 ± 37 nm; data values corresponded to mean ± standard deviation, n = 108.

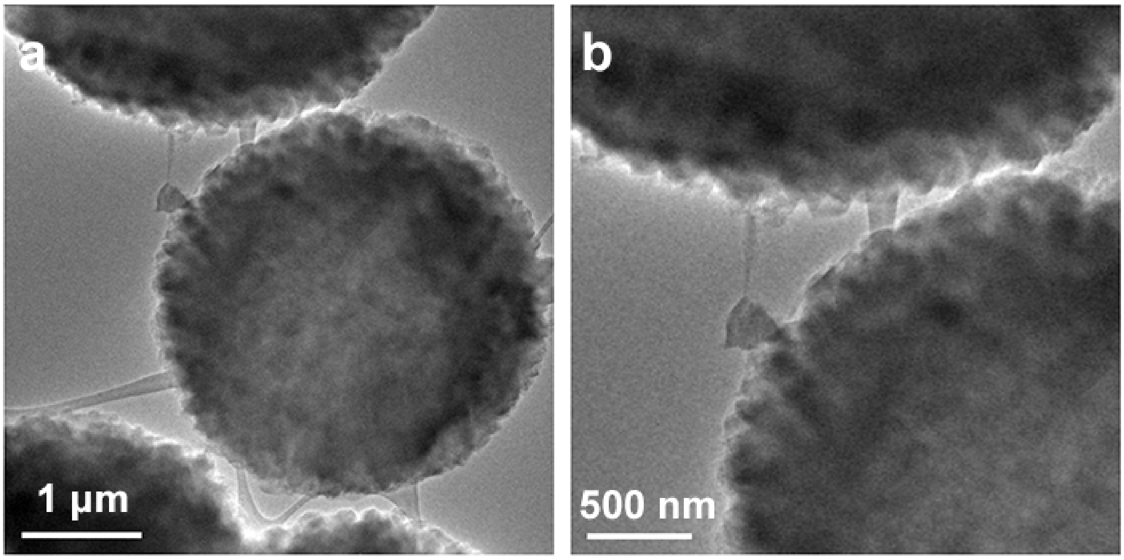

Supplementary Figure 3. Low-magnification TEM image of spherical mCHA zeolite on the cotton.

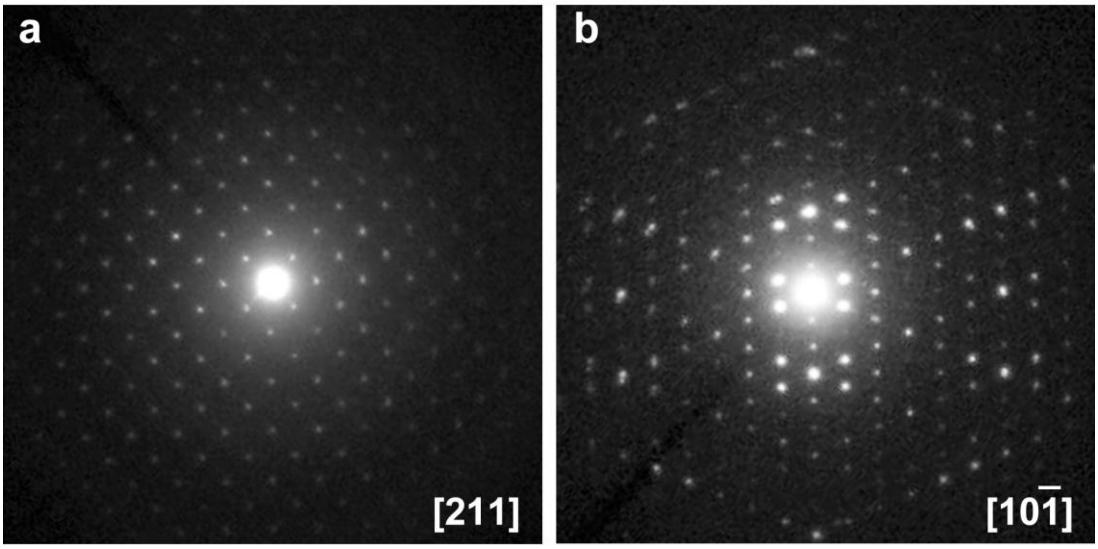

Supplementary Figure 4. Electron diffraction patterns of individual CHA crystal taken along the **(a)** [211] and **(b)** [10$\bar{1}$] zone axes, respectively.

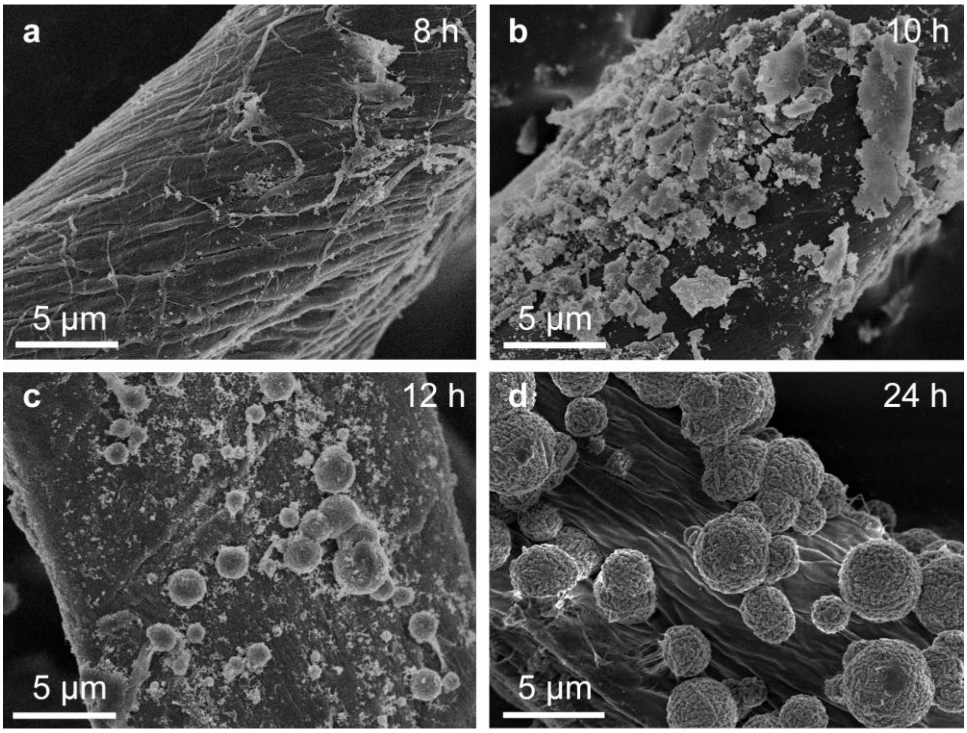

Supplementary Figure 5. FE-SEM images of mCHA-C samples at different time intervals.

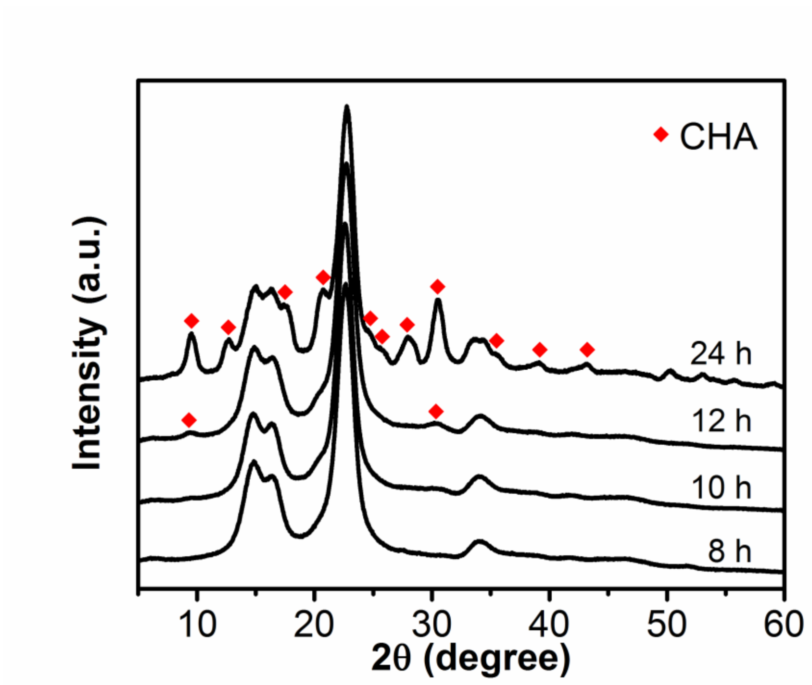

Supplementary Figure 6. XRD patterns of mCHA-C samples at different time intervals. Source data are provided as a Source Data file.

Supplementary Table 3

The solid contents on the cotton surface of mCHA-C samples at different time intervals.

**Time Points**	**8 h**	**10 h**	**12 h**	**24 h**
Solid content (wt%) [#]	5.4	10.2	10.7	23.5

[#] The solid content was measured by TGA.

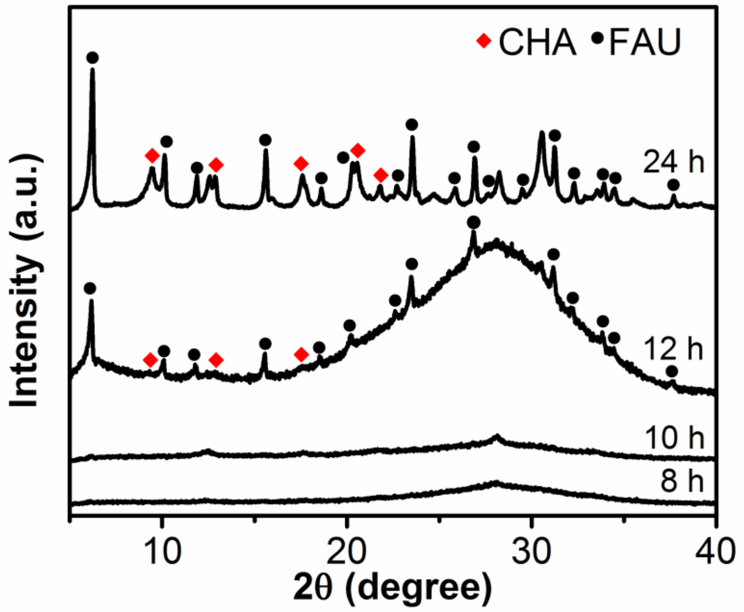

Supplementary Figure 7. XRD patterns of the in-solution products at different time intervals. Source data are provided as a Source Data file.

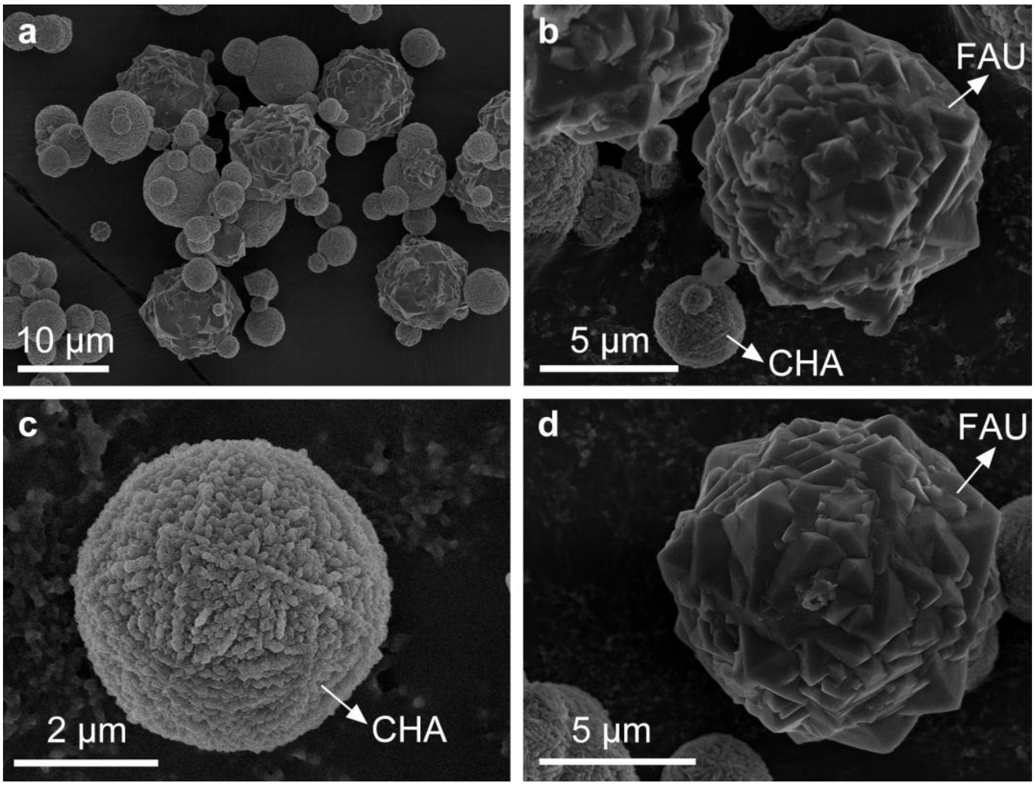

Supplementary Figure 8. (a, b) FE-SEM images of in-solution products in the mCHA-C synthesis. High-magnification FE-SEM images of **(c)** CHA and **(d)** FAU in solution products. CHA zeolite in solution was spherical aggregates, while another solution product was highly-crystalline and bulk FAU zeolite, of which the size was ca. 10 μm.

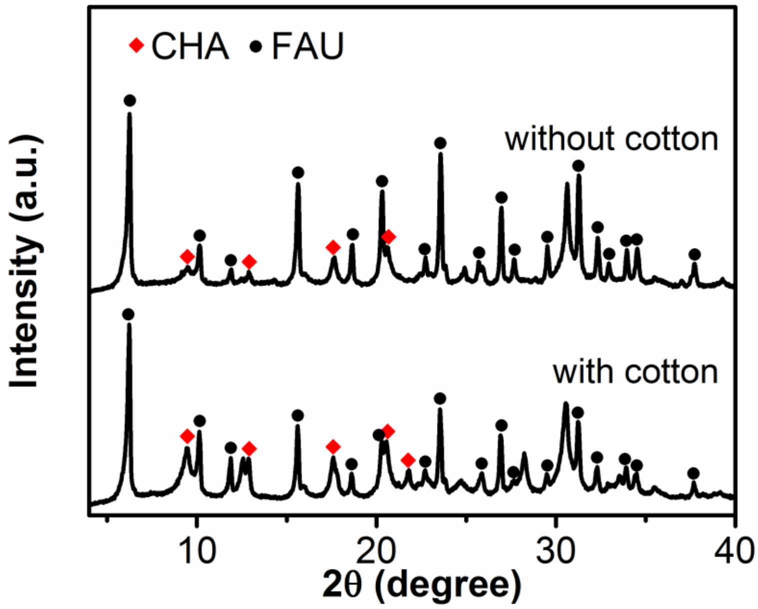

Supplementary Figure 9. XRD patterns of in-solution products with and without cotton. Source data are provided as a Source Data file.

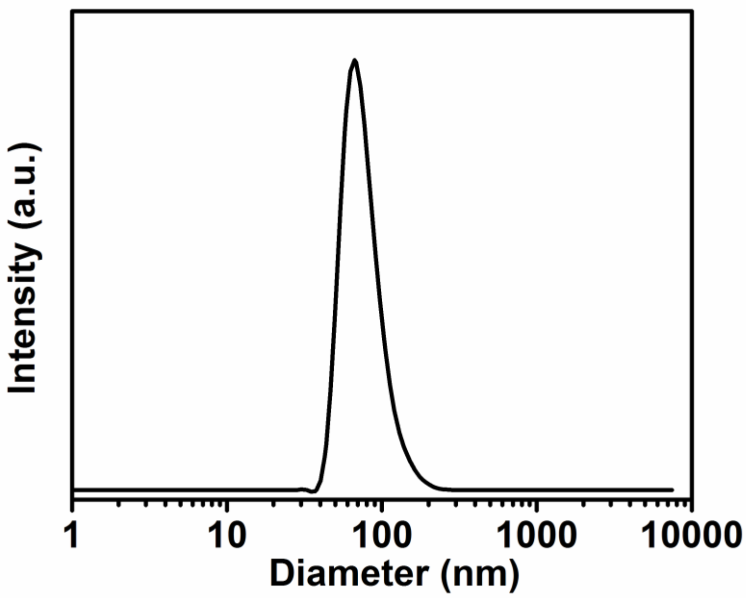

Supplementary Figure 10. DLS curve for the gel particles synthesized at room temperature for 24 h. Source data are provided as a Source Data file.

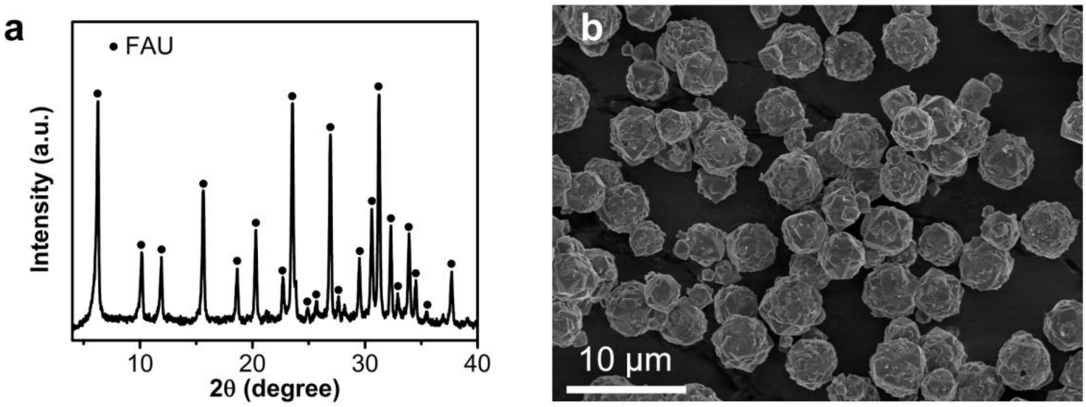

Supplementary Figure 11. (a) XRD pattern and **(b)** FE-SEM image of in-solution product from adding cotton after formation of gel particles. Source data are provided as a Source Data file.

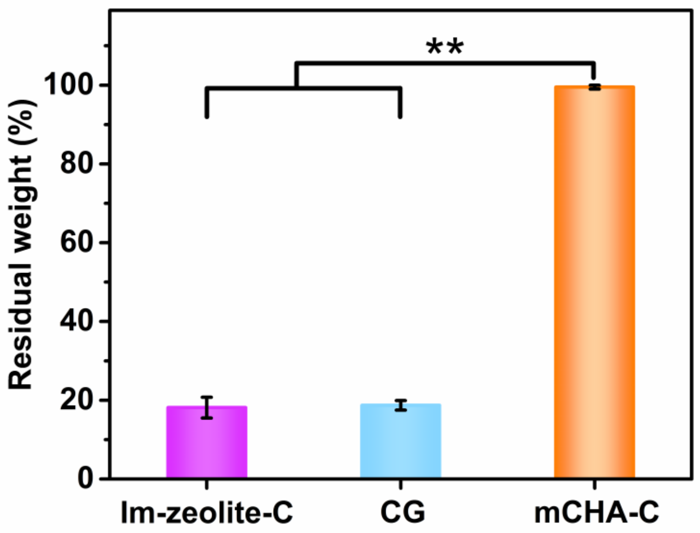

Supplementary Figure 12. The relative residual weight of hemostatic component after washing with deionized water. Source data are provided as a Source Data file. Data values corresponded to mean ± standard deviation, n = 3. Error bars represent standard deviation. **P < 0.01, one-way analysis of variance (ANOVA).

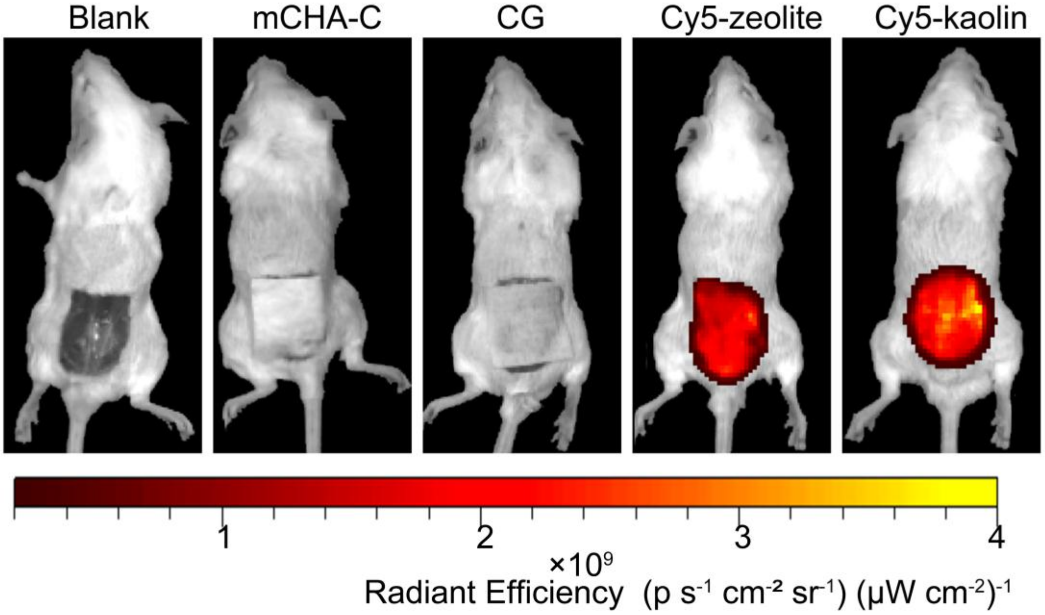

Supplementary Figure 13. Fluorescence imaging of mice skin wound during being treated with mCHA-C, CG, Cy5-zeolite (Cy5-labeled zeolite) and Cy5-kaolin (Cy5-labeled kaolin), respectively.

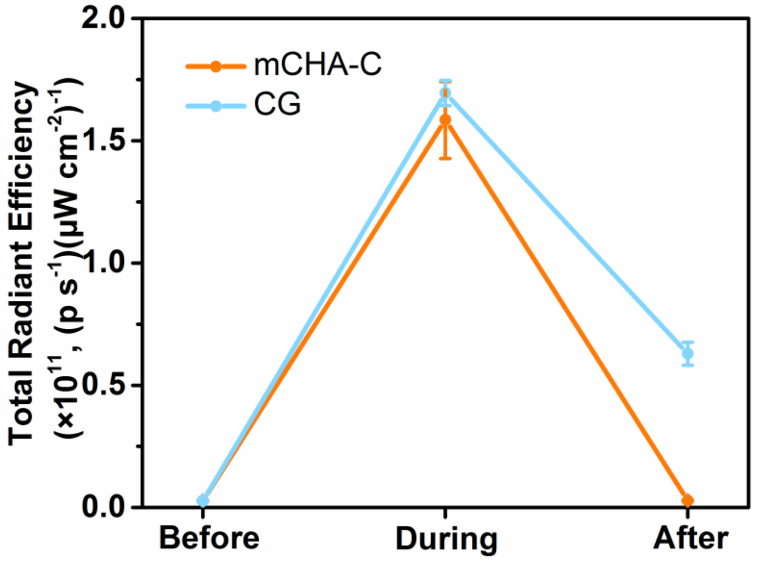

Supplementary Figure 14. Total fluorescent radiant efficiency of mice skin wound before, during and after being treated with Cy5-labeled mCHA-C (orange) and CG (turquoise), respectively. Source data are provided as a Source Data file. Data values corresponded to mean ± standard deviation, n = 3. Error bars represent standard deviation.

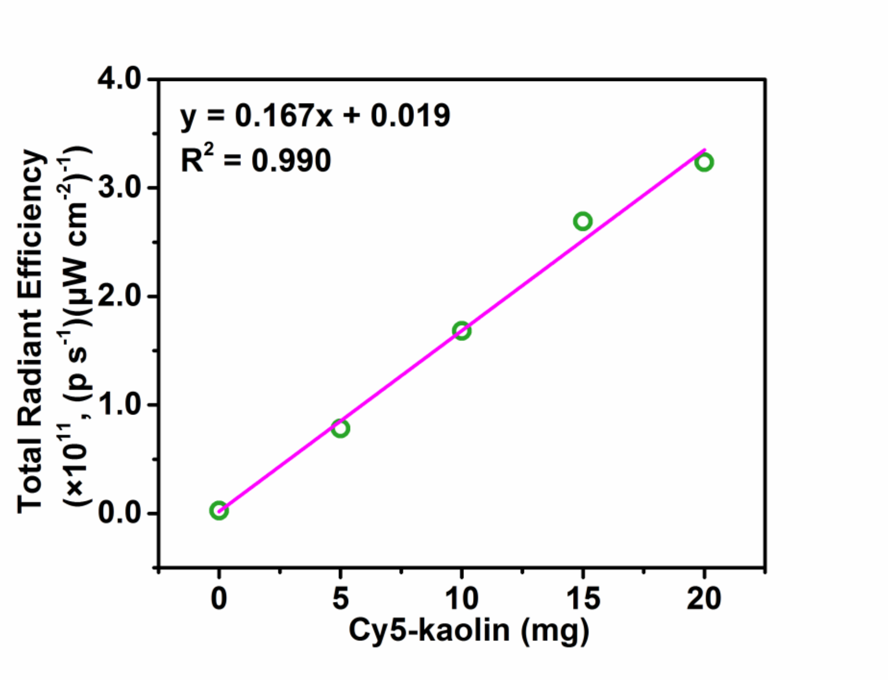

Supplementary Figure 15. Working curve for total fluorescent radiant efficiency of mice skin wound used with Cy5-labeled kaolin. Source data are provided as a Source Data file.

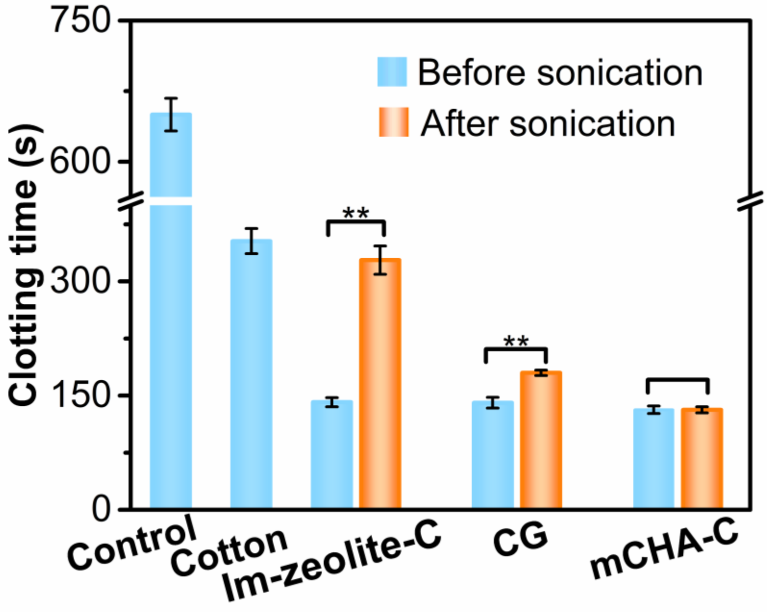

Supplementary Figure 16. Plasma clotting time of hemostats before and after 10 min sonication. Source data are provided as a Source Data file. Data values corresponded to mean ± standard deviation, n = 3. Error bars represent standard deviation. **P < 0.01, one-way analysis of variance (ANOVA).

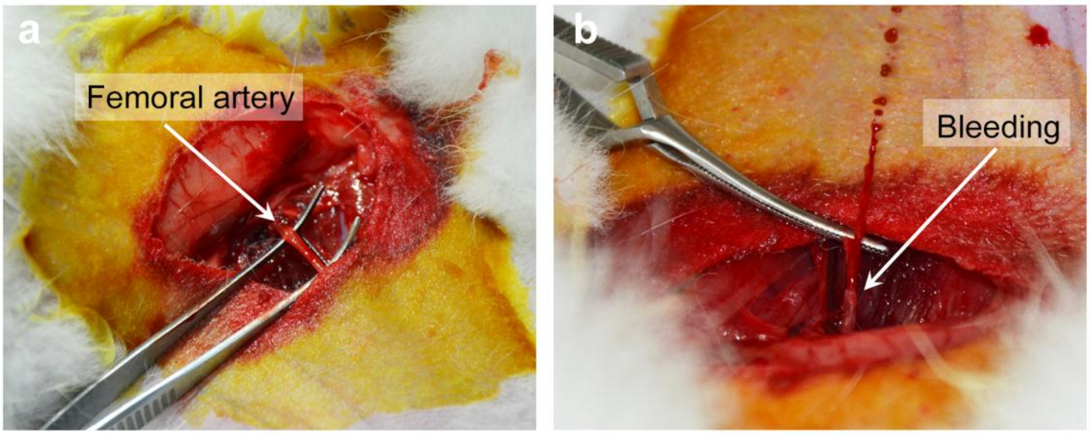

Supplementary Figure 17. The femoral artery was **(a)** exposed and **(b)** transected to make fatal blood loss in the rabbit femoral artery injury model.

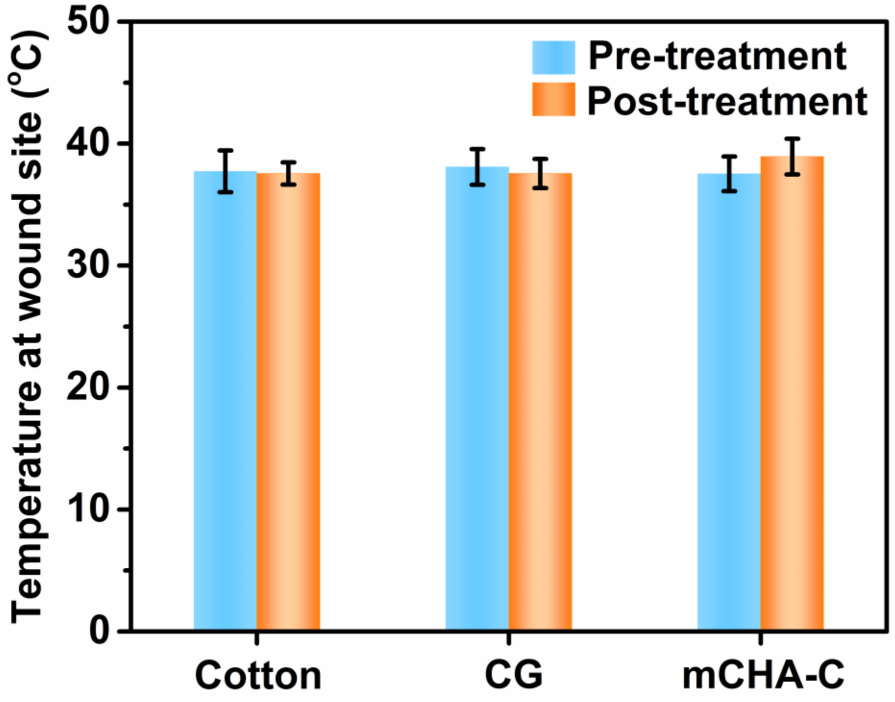

Supplementary Figure 18. Thermal effect of cotton, CG and mCHA-C in the rabbit femoral artery injury model. Source data are provided as a Source Data file. Data values corresponded to mean ± standard deviation, n = 8. Error bars represent standard deviation.

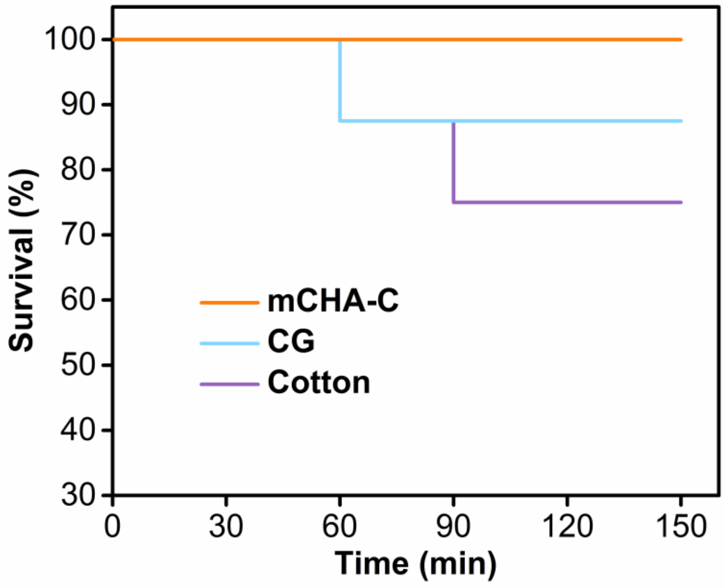

Supplementary Figure 19. Survival analysis of rabbits treated with mCHA-C (orange), CG (turquoise) and cotton (violet) in the fatal femoral artery injure model. Source data are provided as a Source Data file.

Supplementary Table 4

The mCHA content on T-shirt.

Sample	1	2	3	Mean	Standard Deviation
mCHA content (wt%) [#]	22.2	21.6	21.2	21.7	0.5

[#] The mCHA content of mCHA/T-shirt was measured by TGA.

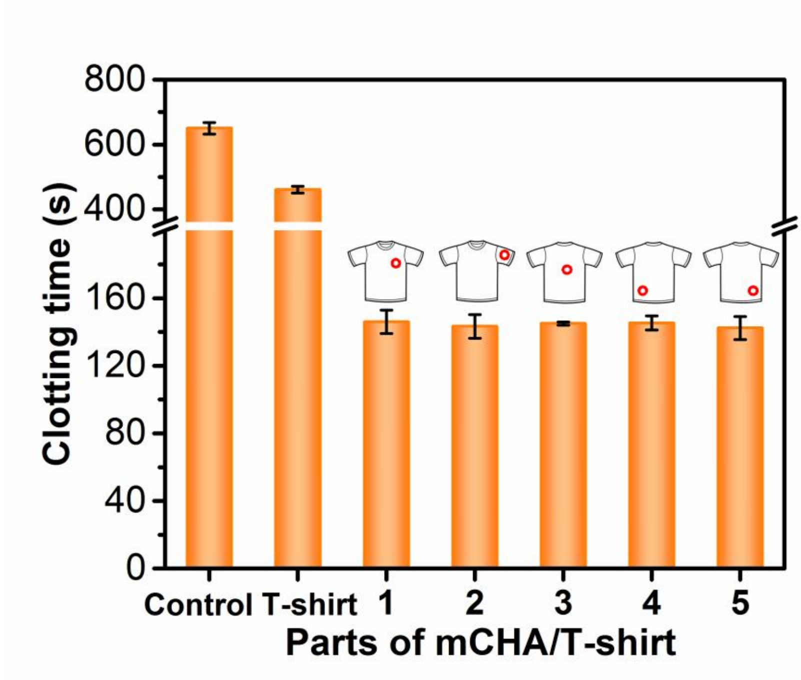

Supplementary Figure 20. The plasma clotting time of five parts of mCHA/T-shirt. Source data are provided as a Source Data file. Data values corresponded to mean ± standard deviation, n = 3. Error bars represent standard deviation.

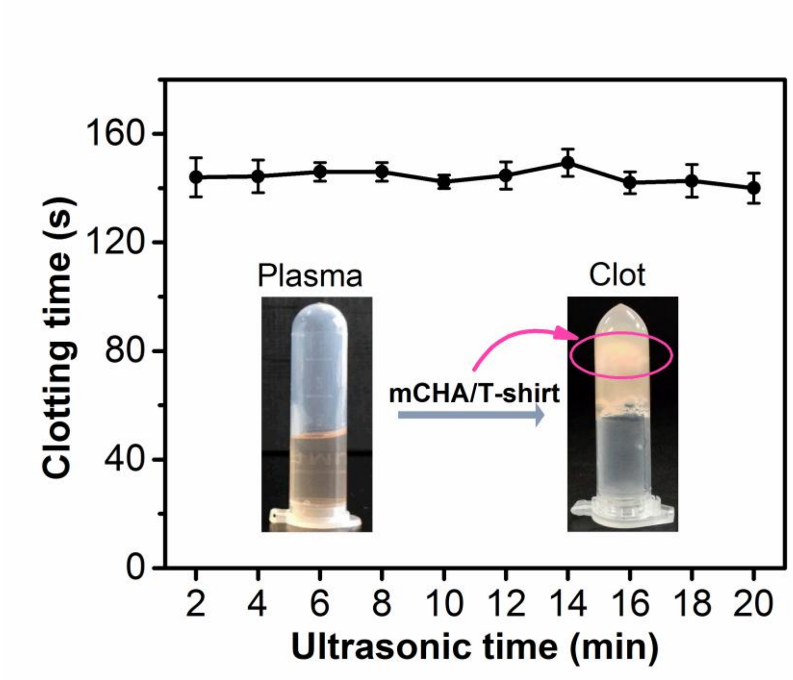

Supplementary Figure 21. The plasma clotting time of mCHA/T-shirt after different ultrasonic time. Source data are provided as a Source Data file. Data values corresponded to mean ± standard deviation, n = 3. Error bars represent standard deviation.
